# Optimization of High-Pressure-Assisted Extraction of Cadmium and Lead from Kelp (*Laminaria japonica*) Using Response Surface Methodology

**DOI:** 10.3390/foods11071036

**Published:** 2022-04-02

**Authors:** Hao Wang, Qiang Wang, Jiahong Zhu, Guixian Hu

**Affiliations:** Institute of Agro-Product Safety and Nutrition, Zhejiang Academy of Agricultural Sciences, 298 Deshengzhong Road, Hangzhou 310021, China; 11513008@zju.edu.cn (H.W.); wq13575733860@126.com (Q.W.); zjnky2011@126.com (J.Z.)

**Keywords:** high-pressure-assisted extraction, kelp, cadmium, lead, response surface methodology

## Abstract

Kelp (*Laminaria japonica*) is a popular and nutritious sea vegetable, but it has a strong biosorption capacity for heavy metals. The high content of cadmium (Cd) and lead (Pb) is a threat to the quality of kelp. The objective of this study was to investigate the effects of high-pressure-assisted extraction (HPAE) conditions on Cd and Pb removal efficiency from kelp. Pressure intensity (0.1–200 MPa), the number of HPAE cycles (one to five) and acetic acid concentration (0–10%) were optimized using response surface methodology. The pressure intensity had the most significant positive effects on Cd and Pb removal efficiency, while the correlation between acetic acid concentration and removal efficiency was positive for Cd and negative for Pb. The optimum conditions for the removal of Cd and Pb were attained at 188 MPa, with four cycles and with an acetic acid concentration of 0%. At optimum conditions, the experimental values of removal efficiency were 61.14% (Cd) and 70.97% (Pb), and this was consistent with the predicted value, confirming the validity of the predictive model.

## 1. Introduction

As a sea vegetable, kelp (*Laminaria japonica*) is a very popular food in people’s daily diet, especially in East Asia. In China, the mariculture area of kelp is about 44,000 hectares, and the output is about 1.49 million tons per year, accounting for 66.5% of the total production of cultured algae. Kelp contains various interesting nutrients that contribute to health benefits. The polysaccharides in kelp perform many biological activities, including anticoagulant [1], hypoglycemic [2], immunostimulatory [3] and antibacterial [4] activities. Kelp is rich in various minerals, especially iodine, an essential trace element for the synthesis of thyroid hormones in the human body [5]. In addition, kelp is also considered to be a good source for the supplemental intake of potassium (69.88 mg/kg DM), sodium (22.97 mg/kg DM), iron (mg/kg DM), magnesium (6.39 mg/kg DM), selenium (0.12 mg/kg DM) and zinc (58.30 mg/kg DM) [6].

Due to heavy metal pollution in the ocean, heavy metal residues in seafood have always been the focus of food safety. Fan et al. [7] reported that the heavy metal pollution of algae was more serious than that of other seafood (marine fish, marine crustaceans and marine soft-bodied animals), and cadmium (Cd) and lead (Pb) are the main pollutants. Cd is mainly stored in the liver and kidney after ingestion. Excessive Cd intake can lead to glomerular damage, kidney failure [8], oxidative stress and the apoptosis of liver cells [9]. The brain and kidney are the main parts affected by Pb toxicity, and excessive exposure to Pb results in neurological, cardiovascular, hematologic and reproductive disturbances of body function [10]. Among macroalgae, the Cd biosorption capacity of *L. japonica* and *Sargassum thunbergii* is higher than that of *Ulva pertusa*, *Enteromorpha linza* and *Chondrus ocellatus* [11]. Xiao et al. [12] studied the biosorption of bivalent metal ions onto *L. japonica* using the bidentate adsorption model and found that the bidentate binding constants for Pb^2+^ was 10 times higher than for Cd^2+^. Due to the strong biosorption of heavy metals in water, kelp has even been studied for the enrichment of Cd and Pb to purify the water environment [13,14]. However, from the perspective of food safety, it is urgent and necessary to remove toxic heavy metals from kelp efficiently.

Several technologies have been applied to remove heavy metals from food. Huo et al. [15] decreased the Cd concentration of rice protein isolate by washing the rice with various acidic solutions. Yang et al. [16] removed the heavy metals (including Cd and Pb) from *Porphyra haitanensis* using 28 kinds of natural deep eutectic solvents. In addition, the application of appropriate assisted extraction technologies can further improve the removal efficiency. High-pressure-assisted extraction (HPAE) is a novel green processing technology [17]. HPAE processes foods at pressures far above atmospheric pressure, so it can increase the diffusion efficiency of the solvent into material cells and the mass transfer efficiency of the extract into the solvent. Moreover, as a nonthermal technology, HPAE has little negative impact on the nutritional and sensory qualities of foods, so it has been applied to extract active ingredients and/or remove pollutants from various foods, including seaweed [18]. Heavy metal removal from foods is a relatively new application field of HPAE. Luo et al. [19] preliminarily explored the Cd removal effect of HPAE on rice grain and rice flour, and the removal efficiency were 43% and 82%, respectively, at optimized conditions. After multiple HPAE cycles, the Cd removal efficiency further improved. Beyond that, no other reported study has researched the removal effect of HPAE on heavy metals in foods.

Therefore, the objective of this study was to preliminarily evaluate the effects of HPAE conditions (pressure intensity, number of cycles and acetic acid concentration) on the removal efficiency of Cd and Pb from kelp. Response surface methodology (RSM) was used to optimize the extraction conditions for the highest removal efficiency.

## 2. Materials and Methods

### 2.1. Materials

The samples of kelp (12.5% moisture content) were obtained from a local supermarket. The kelp was vacuum-sealed in a polyethylene pouch and stored at 4 °C for 24 h.

### 2.2. Experimental Design

A three-level, three-factor Box–Behnken experimental design was used to investigate the effect of the parameters of the HPAE on the removal efficiency of Cd and Pb from kelp (Table 1). The independent variables (factors) were pressure intensity (X_1_, 0.1–200 MPa), number of cycles (X_2_, 1–5 cycles) and acetic acid concentration (X_3_, 0–10%, *v*/*v*). The range and conditions of HPAE were determined according to a previous study [19] and actual production costs. The dependent variables (responses) were the extraction efficiency of Cd (Y_1_) and Pb (Y_2_). The complete design consisted of seventeen randomized trials with five replications at the center.

The responses were assumed to be related to the independent variables by a second-degree polynomial using Equation (1) below:

(1) $$Y_{n}=\beta_{n0}+\overset{3}{\underset{i=1}{\sum }}\beta_{\mathrm{ni}}X_{i}+\overset{3}{\underset{i=1}{\sum }}\beta_{\mathrm{nii}}X_{i}^{2}+\overset{2}{\underset{i=1}{\sum }}\overset{3}{\underset{j=i+1}{\sum }}\beta_{\mathrm{nij}}X_{i}X_{j}$$

where Y_n_ is the response, β_n0_, β_ni_, β_nii_ and β_nij_ are the coefficients of the intercept, linear, quadratic and interaction terms, respectively, and X_i_ and X_j_ are independent variables.

### 2.3. HPAE

The HPAE was performed in a laboratory-scale high-pressure chamber (UHPF-750, Kefa, Baotou, China) with a maximum capacity of 5 L and a potential maximum operating pressure of 750 MPa. Water was used as the pressure transmission medium. The pressure increase rate was about 150 MPa/min, and the depressurization time was less than 10 s.

For HPAE, 5 g samples of kelp were first vacuum-sealed in polyethylene pouches (6 cm × 8.5 cm) together with 125 mL of acetic acid solution (0%, 5% and 10%, *v*/*v*, diluted in distilled water). For each HPAE cycle, these pouches were incubated at 25 °C for 30 s, and then transferred to the high-pressure chamber for pressurization (0.1, 100.05 and 200 MPa). The pressure holding time was 0 s; in other words, the pressure was released immediately when the pressure reached the set value. Normally, the sample temperature is expected to increase by about 3 °C for every 100 MPa pressure rise because of adiabatic compression [20]. However, because of heat loss to the thick-walled stainless steel pressure vessel, this increase was minimal (~2 °C) and was not considered to be significant for the tests performed below 200 MPa. The pressure vessel was not influenced by the adiabatic compression heating of the chamber contents and remained at relatively the same temperature and, thus, could absorb the heat from the sample and water.

After cycles of HPAE, the samples were washed with deionized water and then dried in an air oven at 30 °C until the average moisture content was about 12.5 ± 0.5% (wet basis). All experiments were conducted in triplicate.

### 2.4. Determinaton of Cd and Pb via Inductively Coupled Plasma–Mass Spectrometry (ICP-MS)

The Cd and Pb concentrations of all samples were determined via ICP-MS according to Deng et al. [21], with some modifications. Firstly, the kelp samples were ground and sieved through a 60-mesh screen, and then 0.1 g (exact to 0.0001 g) of finely ground kelp was weighed into a polytetrafluoroethylene (PTFE) container, predigested with 6.5 mL of 68% (*w*/*w*) HNO_3_ and 0.5 mL of 40% (*w*/*w*) HF at 120 °C for 30 min in a graphite heater (G400, PreeKem, Shanghai, China). Secondly, samples were further digested in a microwave digestion system (TOPEX+, PreeKem, Shanghai, China) under a stepwise temperature-controlled program: the initial temperature of 120 °C was maintained for 2 min, then raised to 150 °C, maintained for 2 min, and then raised to 180 °C, maintained for 2 min, and finally increased to 200 °C and maintained for 20 min. Then, digested samples were heated at 180 °C for 30 min in a graphite heater to drive the residual acids. After cooling, the digested solutions were diluted to 25 mL with deionized water. The Cd and Pb concentrations in the digestion were analyzed using an ICP-MS instrument (Agilent 8900 ICP-MS/MS, Agilent Technologies, Santa Clara, CA, USA) in helium (He) mode. The ICP-MS operating parameters were set as follows: RF forward power 1550 w, carrier gas flow rate 1.05 L/min, dilution gas flow rate 0.15 L/min, He cell gas flow rate 4.5 L/min, nebulizer type MicroMist and sample uptake rate 0.4 mL/min. A standard solution containing ^111^Cd and ^206^Pb and internal standards (^72^Ge and ^185^Re) were obtained from Guobiao Testing & Certification Co., Ltd., and used for calibration.

The Cd and Pb removal efficiency were calculated using Equation (2) below:

(2) $$\mathrm{RE}=\frac{C_{0}m_{0}-C_{1}m_{1}}{C_{0}m_{0}}\times 100\%$$

where RE is the removal efficiency of heavy metals (%), $m_{0}$ and $m_{1}$ are the masses of kelp before and after the HPAE treatment, respectively. $C_{0}$ (μg/g) and $C_{1}$ (μg/g) are the concentrations of heavy metals in kelp before and after the HPAE treatment, respectively.

### 2.5. Statistical Analysis

The analysis was performed individually for all the responses. The multiple regression analysis was performed with Design-Expert software (version 12.0.3.0, Stat-Ease, Inc., Minneapolis, MN, USA) to fit a second-degree polynomial model including linear, quadratic and interaction terms for the independent variables, and to determine the β coefficients. For model analysis, non-significant factor terms (*p* > 0.05) were eliminated from the initial model, unless a quadratic or interaction effect including that factor were significant. For quadratic or interaction terms, *p*-values greater than 0.10 indicated that the model terms were not significant, and they were eliminated from the initial model. After reduction, the model was fitted to the experimental data. The efficiency of the model was investigated by determining the *p*-value of the regression equation, the number of significant terms, the *p*-value of the lack of fit test and the coefficients of determination (R^2^) and adjusted R^2^ [22,23]. Three-dimensional (3D) response surface plots were designed for significant (*p* < 0.05) interactions. Numerical optimization was performed using a desirability function [23] to predict the optimum level of independent variables providing the highest removal efficiency of Cd and Pb. Experiments at optimum conditions were carried out with three replications in order to validate the models developed by comparing the experimental data with the predicted values. A single-sample *t*-test (*p* < 0.05) was applied to compare the differences between predicted value and experimental value using SPSS 20.0 (SPSS Inc., Chicago, IL, USA).

## 3. Results and Discussion

### 3.1. Effect of HPAE Conditions on Cd Removal Efficiency

Using the experimental data in Table 2, the model of Cd removal efficiency was fitted initially. In the initial model, the quadratic terms of cycles and acetic acid concentrations and all interaction terms were not significant, so they were reduced. Finally, the β regression coefficients of significant terms and the investigation results of model efficiency were determined as listed in Table 3. The reduced model was statistically significant (*p* < 0.0001). The R^2^ and adjusted R^2^ were 0.9848 and 0.9798, respectively. The R^2^ being close to unity and the adjusted R^2^ being close to the R^2^ ensured the satisfactory fitting of the model to the real system [24]. The lack of fit measures the failure of the model to represent the data in the experimental domain at points which are not included in the regression [25]. A non-significant lack-of-fit was considered to be desirable. The value of the lack of fit for the reduced regression model was not significant at the 5% level (*p* = 0.1126 > 0.05), indicating the good predictability of the model.

As shown in Table 2, the HPAE removal efficiency of Cd from kelp ranged from 20.29 to 67.48% depending on the HPAE conditions. Within the designed experimental conditions in the present study, the maximum removal efficiency corresponded to the removal of Cd at 200 MPa for three cycles with 10% acetic acid solution. The Fisher F-test and the probability (*p*) values serve as a tool to check the significance of each of the variables. Pressure intensity, number of cycles, acetic acid concentration and the quadratic term of pressure intensity were significantly correlated with the Cd removal efficiency (*p* < 0.0001, Table 4). In addition, the larger F-ratio and smaller *p*-value mean the corresponding variable was more significant [22,26]. Therefore, pressure intensity had the most significant effects on Cd removal efficiency from kelp. Fernandes et al. [27] also found a lower *p*-value for pressure intensity than those of extraction time and solvent concentration after the optimization of HPAE flavonoids and anthocyanins from pansies. The high pressure could improve the ability of the solvent to permeate into the material and extract the target components, and a dissolution equilibrium could be achieved in a very short time [19]. Generally, within a certain pressure range, the higher the pressure, the higher the extraction efficiency. In this study, the Cd removal efficiency ranged from 38.13 to 61.79% at 100.05 MPa and 54.98 to 67.48% at 200 MPa. Similar results were also found below 200–300 MPa by He et al. [28], who applied HPAE to phenolic acid extraction from *Deodeok*. However, they also reported that when the pressure increased further (>300 MPa), the increase in the extraction efficiency was not significant. In the present study, the quadratic term of pressure intensity was significant and negative, confirming that the effect of pressure intensity on the improvement of removal efficiency would be weaker at a higher pressure level. There was also no significant difference in Cd removal efficiency from rice grain among extraction pressures at 300, 450 and 600 MPa, while multiple cycles of HPAE increased Cd removal efficiency from 48% (one cycle) to 94% (four cycles) [19]. Similarly, the number of HPAE cycles had a significant effect on Cd removal efficiency from kelp in the present study. Instantaneous decompression results in a temporary and large pressure difference between the inside and outside of the samples. Therefore, there is a rapid outflux of compressed solvent during the release of pressure, causing the destruction of the outer structure of samples and an increase in permeability [29]. The more cycles of high pressure, the better the permeability. There was a positive correlation between acetic acid concentration and Cd removal efficiency, which could be attributed to the ion exchange between H^+^ and Cd^2+^ in kelp [7]. The acidic acetic acid solution first permeates into the interior of the matrix and then extracts the Cd from the kelp. Due to the concentration difference in Cd between the interior kelp matrix and the external solvent, Cd eventually diffused out of the kelp. The solvent with a higher concentration of acids was more effective because of the better solubility of Cd [19].

### 3.2. Effect of HPAE Conditions on Pb Removal Efficiency

After the initial fitting of the model of Pb removal efficiency, we found that all terms were significant (*p* < 0.05) and, therefore, retained them. As shown in Table 3, the model was statistically significant (*p* < 0.0001) with no significant (*p* = 0.1156 > 0.05) lack of fit, indicating that the model adequately described the relationship between the Pb removal efficiency and HPAE conditions (pressure intensity, number of cycles and acetic acid concentration). The R^2^ and adjusted R^2^ were 0.9949 and 0.9884, respectively, indicating a satisfactory fitting of the quadratic models to the experimental data and good correlation between the experimental and predicted values.

The HPAE removal efficiency of Pb from kelp ranged from 6.82 to 63.36% depending on the HPAE conditions. Within the designed experimental conditions in the present study, the maximum removal efficiency corresponded to the removal of Cd at 200 MPa for three cycles with 0% acetic acid solution. As with the result for Cd, the pressure intensity had the largest F-ratio, and both pressure intensity and the number of HPAE cycles had a positive effect on Pb removal efficiency. Differently, the β_3_ in the Pb removal model was −8.57, indicating that the Pb removal efficiency was lower when using a solvent with a higher acetic acid concentration. Bo et al. [30] optimized the extraction conditions of heavy metals in food packaging inner lining paper using RSM, and also demonstrated that the β_i_ values of acetic acid concentrations were −0.025 (Pb), 0.02075 (As), 0.01862 (Cd) and 0.19625 (Cr) in the model. This phenomenon was likely due to the fact that lead acetate ((CH_3_COO)_2_Pb) is a weak electrolyte, which does not completely ionize in a solution. After being extracted by acids, other heavy metals were present in the solvents as ions, such as Cd^2+^, while some of the Pb was present in a molecular form (lead acetate). In general, molecules are less diffusible than ions. Therefore, more acetate (CH_3_COO^−^) permeating into kelp resulted in Pb being harder to be extracted.

**Figure 1:**
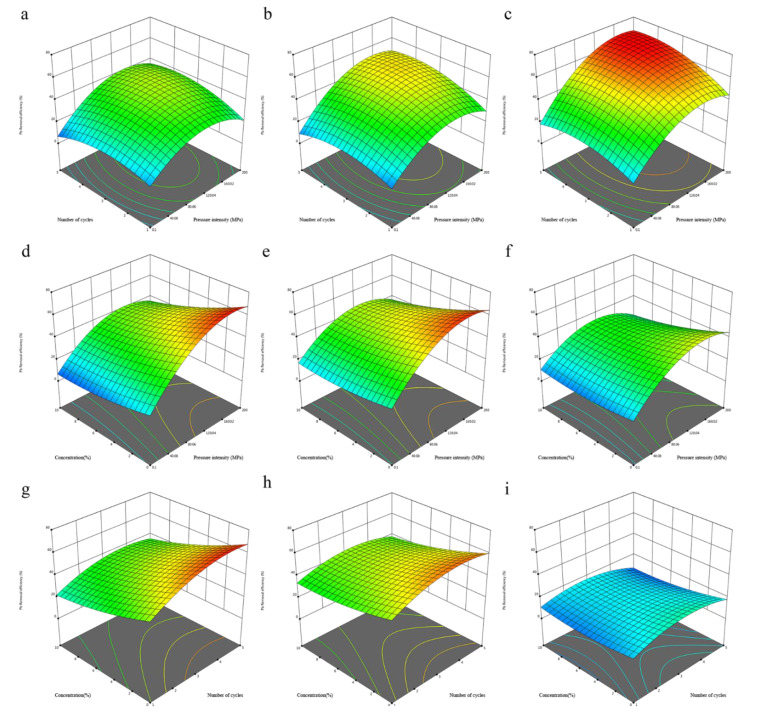
Three-dimensional surface plots showing interaction effects of independent variables on the Pb removal efficiency: (**a**) pressure intensity–number of cycles, effect at concentration of 10%; (**b**) pressure intensity–number of cycles, effect at concentration of 5%; (**c**) pressure intensity–number of cycles, effect at concentration of 0%; (**d**) pressure intensity–concentration, effect at 5 cycles; (**e**) pressure intensity–concentration, effect at 3 cycles; (**f**) pressure intensity–concentration, effect at 1 cycle; (**g**) number of cycles–concentration, effect at 200 MPa; (**h**) number of cycles–concentration, effect at 100.05 MPa; (**i**) number of cycles–concentration, effect at 0.1 MPa.

All interaction terms were significant, so the 3D response surface plots for the three independent variables were generated by keeping the one independent variable as the experimental value, as shown in Figure 1a–i. The interaction effect between the pressure intensity and number of cycles showed a positive effect on the Pb removal efficiency. Although the pressure holding time in this study was 0 s, it still meant that the samples were in a condition of higher than atmospheric pressure for more time after being pressurized for more cycles. Therefore, the effect of pressure intensity on solvent permeability was more pronounced. Meanwhile, the temporary pressure difference between the inside and outside of the kelp during decompression was larger when a higher pressure was applied. According to Figure 1a,b, an increase in pressure up to 160 MPa resulted in higher Pb removal efficiency, but beyond 160 MPa, a slight drop occurred. HPAE with four cycles provide a higher Pb removal efficiency than less or more cycles. However, the decrease was not obviously observed in Figure 1c. Therefore, this slight decrease at a higher pressure with more cycles could be explained by more acetate being present inside the kelp. On the one hand, the dissociation of acetic acids will be increased under high pressure, generating more acetate [31]. On the other hand, higher pressure and multiple HPAE cycles provide more force for the permeation of acetate, and 160 MPa with four cycles might be the threshold for a great increase in the permeability of acetate. Figure 1d–f and Figure 1g–i showed the interaction of X_1_X_3_ and X_2_X_3_, respectively. The increase in the Pb removal efficiency induced by a higher pressure or multiple cycles was more pronounced when using a low concentration of acetic acid. In the present study, all quadratic coefficients (β_ii_) were the opposite of the corresponding linear coefficients (β_i_). A similar phenomenon has been reported in previous studies on the optimization of HPAE conditions using RSM [32,33]. This result indicated that all factors had dual positive and negative effects on Pb removal efficiency. The positive effect was mainly due to the ion exchange between hydrogen ions and Pb, while the negative effect was induced by the generation of lead acetate. Therefore, it is necessary to optimize the extraction conditions to achieve the maximum removal efficiency.

### 3.3. Optimization of HPAE Conditions and Validation of the Models

Numerical optimization was realized using the desirability function to obtain the optimum HPAE conditions with the highest Cd and Pb removal efficiency. The initially calculated optimum HPAE using Design Expert was attained at 188.009 MPa, with 4.99995 cycles and an acetic acid concentration of 2.55 × 10^−6^%, which were simplified as 188 MPa, five cycles and an acetic acid concentration of 0%. As shown in Table 5, the simplified predicted values of the Cd and Pb removal efficiency were 59.49% and 68.58%, respectively, the same as the initially predicted values. Therefore, the adequacy of the predictive models at the simplified optimum condition was validated by performing three independent experiments. The experimental values of the Cd and Pb removal efficiency were 60.47% and 67.09%, respectively, and there was no significant difference between the predicted values and experimental values (*p* > 0.05), confirming the validity of the optimum HPAE conditions. In a previous study, the optimum conditions of the conventional soaking extraction of heavy metals from kelp was reported at pH 2.0, with an extraction time of 4 h and a solvent/sample ratio of 125:1 (mL/g), which could remove 61.14% of Cd and 70.97% of Pb [7]. By contrast, HPAE can achieve the efficient removal of heavy metals in a shorter time and with less solvent.

## 4. Conclusions

The present study investigated the effects of high-pressure-assisted extraction conditions (pressure intensity, number of cycles and acetic acid concentration) on Cd and Pb removal efficiency from kelp using response surface methodology. After optimizing the models by reducing non-significant terms, the predictive models were adequate for describing the relationship between the factors and responses. Among these variables, pressure intensity was the most significant variable (with the largest F-ratio). For Cd, a higher pressure, more cycles and a higher acetic acid concentration were more conducive to removal. For Pb, the 3D response surface plots revealed that extraction at ~160 MPa, with four cycles using a low-concentration acetic acid solution was the most desirable condition for high efficiency. The optimum conditions for Cd and Pb removal was simplified as 188 MPa, with five cycles and an acetic acid concentration of 0%, which could achieve 60.47% and 67.09% removal efficiency, respectively. This study demonstrated that high-pressure-assisted extraction has a promising application in the field of removing multiple heavy metals from food and has the advantages of high efficiency and good economy compared with conventional extraction methods. In addition, future studies should further focus on the important nutrients lost in HPAE-treated kelp, and set the overall optimum parameters of HPAE to achieve the highest removal efficiency of harmful heavy metals and the lowest extraction efficiency of important nutrients.

## Figures and Tables

**Table 1:** Experimental design, including process variables and their levels expressed in terms of coded and uncoded variables.

Runs	Coded Variables			Uncoded Variables		
	X_1_	X_2_	X_3_	Pressure Intensity (MPa)	Number of Cycles	Acetic Acid Concentration (%)
1	+1	−1	0	200	1	5
2	+1	+1	0	200	5	5
3	−1	−1	0	0.1	1	5
4	0	0	0	100.05	3	5
5	0	+1	−1	100.05	5	0
6	0	+1	+1	100.05	5	10
7	+1	0	+1	200	3	10
8	0	0	0	100.05	3	5
9	0	0	0	100.05	3	5
10	+1	0	−1	200	3	0
11	−1	+1	0	0.1	5	5
12	0	−1	+1	100.05	1	10
13	0	−1	−1	100.05	1	0
14	0	0	0	100.05	3	5
15	−1	0	+1	0.1	3	10
16	0	0	0	100.05	3	5
17	−1	0	−1	0.1	3	0

**Table 2:** Experimental values for removal efficiency using a Box–Behnken design.

Runs	Removal Efficiency (%)	
	Cd	Pb
1	23.35 ± 0.87	6.82 ± 0.05
2	20.29 ± 0.49	25.49 ± 0.90
3	32.12 ± 2.78	18.26 ± 0.92
4	26.90 ± 0.69	8.38 ± 0.37
5	38.13 ± 2.27	46.96 ± 1.14
6	51.29 ± 3.36	34.39 ± 1.97
7	52.87 ± 2.34	49.92 ± 2.94
8	52.16 ± 3.77	49.79 ± 3.94
9	50.22 ± 3.38	51.59 ± 1.62
10	50.52 ± 4.05	49.11 ± 3.27
11	50.33 ± 2.65	48.31 ± 2.04
12	53.84 ± 2.00	59.48 ± 3.12
13	61.79 ± 3.26	37.96 ± 1.70
14	56.88 ± 3.14	30.87 ± 0.29
15	54.98 ± 1.25	63.36 ± 4.92
16	67.48 ± 1.36	36.11 ± 1.67
17	64.10 ± 1.31	50.47 ± 3.25

Values are means ± standard deviations (*n* = 3).

**Table 3:** Regression coefficients of coded factors, lack of fit, R^2^, adjusted R^2^ and *p*-value (regression) for the final reduced models.

Source		Removal Efficiency (%)	
		Cd	Pb
**Regression coefficient**	β_0_	51.24	49.74
	Linear		
	β_1_	17.60	15.23
	β_2_	4.62	4.66
	β_3_	5.68	−8.57
	Quadratic		
	β_11_	−7.98	−17.25
	β_22_	-	−8.36
	β_33_	-	3.31
	Interaction		
	β_12_	-	4.51
	β_13_	-	−5.00
	β_23_	-	−2.24
Lack of fit		0.1126	0.1156
R^2^		0.9848	0.9949
Adjusted R^2^		0.9798	0.9884
*p*-Value (regression)		<0.0001	<0.0001

**Table 4:** *p*-Value and F-ratio of HPAE variables in final reduced models.

Terms	Cd		Pb	
	*p*-Value	F-Ratio	*p*-Value	F-Ratio
Main effects				
$X_{1}$	<0.0001	607.14	<0.0001	572.11
$X_{2}$	<0.0001	41.89	0.0002	53.44
$X_{3}$	<0.0001	63.25	<0.0001	181.27
Quadratic effects				
$X_{1}^{2}$	<0.0001	66.03	<0.0001	386.16
$X_{2}^{2}$	-		<0.0001	90.67
$X_{3}^{2}$	-		0.0070	14.24
Interaction effects				
$X_{1}X_{2}$	-		0.0016	25.07
$X_{1}X_{3}$	-		0.0009	30.86
$X_{2}X_{3}$	-		0.0420	6.17

**Table 5:** Predicted and experimental values of removal efficiency at optimum conditions.

	Removal Efficiency (%)		
	Initial Predicted Values	Simplified Predicted Values	Experimental Values
Cd	59.49	59.49	60.47 ± 2.08
Pb	68.58	68.58	67.09 ± 1.99

Values are means ± standard deviations (*n* = 3).

## Data Availability

The data presented in this study are available on request from the corresponding author. The data are not publicly available due to data confidentiality.
